# Group Psychological Counseling Based Growth Mindset Intervention To Promote Active Aging Behaviors In Older People

**DOI:** 10.1093/geroni/igaf122.814

**Published:** 2025-12-31

**Authors:** Qian Yang, Xiaohua Xiao

**Affiliations:** School of Public Health, Zhejiang University School of Medicine, Hangzhou, Zhejiang, China (People’s Republic); Zhejiang University, Hangzhou, Zhejiang, China (People’s Republic)

## Abstract

Misconceptions that aging leads only to decline and is uncontrollable undermine older people’ autonomy and discourage active aging behaviors. Few studies focus on older people’ autonomy. The growth mindset theory, which posits that abilities can be developed through effort, aligns with the active aging framework and serve as a key intervention target to enhance engagement in active aging behaviors. However, research on growth mindset interventions for older people remains scarce. This study addresses the gap in growth mindset interventions for older people by using group counseling to promote active aging behaviors. A randomized controlled trial design was employed, with an autonomous intervention group receiving four structured counseling sessions and a control group engaging in equivalent community activities. The study was conducted in six communities across eastern and western China. Growth mindset, active health behaviors, social participation, and self-security behaviors were evaluated at four timepoints: baseline (T1), post-intervention (T2), and 1- and 3-month follow-ups (T3/T4). The intervention group showed significant improvements in growth mindset and active social participation behavior at (T2/T3/T4) (B > 0.00, p < 0.00) and demonstrated greater improvement in active health behaviors at T2 compared to the control group (B = 0.12, p < 0.00). The intervention group showed improvements in active self-security behavior at T3 (B = 0.16, p < 0.00). This study is the first to validate the effectiveness of growth mindset interventions tailored to older people, providing a novel approach to enhancing active aging behaviors and addressing the challenges posed by an aging population.

